# Developmental Effects of Davydov’s Mathematics Curriculum in Relation to School Readiness Level and Teacher Experience

**DOI:** 10.3389/fpsyg.2020.603673

**Published:** 2020-11-19

**Authors:** Anastasia Sidneva

**Affiliations:** Department of Psychology, Lomonosov Moscow State University, Moscow, Russia

**Keywords:** mathematics, school readiness, first graders, developmental effects, Cultural Historical Activity Theory (CHAT)

## Abstract

Davydov’s mathematics curriculum was designed according to the principles of the Cultural Historical Activity Theory (CHAT). In this study, we analyzed some developmental effects of its realization in Grade 1 (*N* = 46, two classes), in relation to the children’s school readiness level (specifically, their motivation, voluntary regulation, and intellectual development), and their teacher’s experience (a very experienced teacher in one class, and a less experienced one in the other). We assessed two groups of developmental effects: (1) some general math abilities (comparison of objects, measurement and ability to solve simple problems of addition and subtraction); and (2) some abilities, which are very specific to Davydov’s mathematics curriculum (i.e., the ability to put numbers on a number line and to measure quantity using different measures). At the beginning of the Grade 1, we divided all participants into three groups according to their level of motivation (low, medium, or high), voluntary regulation (low, medium, or high), and logical preservation (low, medium, or high) in terms of J. Piaget. After 1 year (at the beginning of Grade 2) we measured general effects again and also measured specific effects. The results showed that all the children became significantly better in all general math abilities. It was also found that progress in math abilities does not depend on the initial level of school readiness of children. Children with different levels of voluntary regulation, motivational readiness, and the level of logical preservation show improvements in general math abilities. As for the mathematical skills specific to the Davydov’ program, the achieved level of their development (both according to the test results and the results of the expert assessment by the teacher) is also not related to the initial parameters of readiness for school. Also, there were no differences in improvements in general effects and in specific effects between the classes with experienced and not-so-experienced teacher. So, there are some reasons to believe that the level of the child’s actual development does not play a fundamental role for education, which built in accordance with the principles of CHAT.

## Introduction

The Elkonin-Davydov educational system emerged in the Soviet Union more than 60 years ago. It was among others (see, for example, Zankov, 1975; Cole, 1990; Bibler, 1993; Puentes, 2017) the attempt to apply Vygotsky’s main idea about the interconnection between learning and development to education. Vygotsky (1978) suggested absolutely different way for understanding human nature, and, as the result, for understanding of the role of learning and teaching in child development. In contrast to J. Piaget, in whose studies development is considered as a natural process of adaptation to the environment, Vygotsky believed that a “subjective world is born from the objective world of art, from the world of production tools, from the world of industry” (Obukhova, 2012, p. 51), i.e., child development should be considered as cultural development. So, learning and teaching according to Vygotsky is the major factor of cognitive development. If, according to Piaget, learning depends on the level of development achieved by the child, then according to Vygotsky (1956), learning leads development, “bringing to life” those processes that cannot arise without it. Moreover, each subject at school has its own, special relation to the course of child development and it is important for a psychologist to find “the internal structure of school subjects from the point of view of the child’s development and changes in this structure along with the methods of school teaching” (Vygotsky, 1956). So what are these developmental processes that are brought to life by school teaching, in particular, mathematics? Does mathematics curriculum, which was built in Vygotsky’ foundations, can help even students with less school readiness to achieve some key math abilities?

## Elkonin-Davydov’s Developmental Education: General Principles and Composition of Math Curriculum

Elkonin-Davydov approach was called developmental education (DE), because the main goal of such system is to encourage students’ learning initiative, independent critical thinking, and the development of reflective abilities (Zuckerman, 2014). Moreover, such learning has to be organized in a specific way and therefore requires a radical restructuring of both the content and form of classroom learning (Elkonin, 1975; Davydov, 1990; Zuckerman, 2014). The main differences between the content of DE and traditional education (TE) concern, first of all, the type of subject matter: the DE curriculum involves theoretical concepts and the TE-system involves empirical ones (Davydov, 1990). Theoretical knowledge is knowledge about the genesis of the concept being learned. Theoretical knowledge allows children to understand not only how to do something, but also why to perform a task this way and not another. The second point is the way children’s learning activities are organized. It is important to choose actions in which theoretical concepts can assume the role of a cultural mediator (Vygotsky, 1978), a model. It means that students’ aware of the functions of these concepts in the whole system (i.e., in which kind of tasks do people need a number?). The definition of such a function was the goal of a specific action, which was called a “learning task” in El’konin’s and Davydov’s theory. The “generation” of such theoretical concept through solving of learning task should be constructed on the basis of special “logical-genetic” reconstruction of the process by which a concept emerges (Davydov, 1990). So, after an understanding and accepting of learning task the next student’s learning action is the transformation of the situation and discovering of the general foundation for the further solution of a variety of individual problems. Other learning actions are modeling of the general way of actions, their application to different problems, control and assessment. Active and problem-oriented methods of instruction are also widely used in this curriculum, as well as forms of student self-assessment from the first grade on (Davydov, 1990).

The mathematics curriculum is a classical example of the implementation of the Elkonin-Davydov approach to practice of teaching. Most traditional math curricula assume that if children can count when they enter first grade then they “know what number is,” and move on to training the children in operations with number (Moxhay, 2008). Davydov’ mathematics curriculum is structured in this way so the concept of number (as theoretical concept) emerges out of the concept of mathematical quantity as its precondition. One (Gorbov et al., 2008) or two (Aleksandrova, 2001) trimesters of Grade 1 in the DE curriculum focuses on pre-numerical learning. Students study the properties of objects such as color, shape, and size, and then quantities such as length, volume, area, mass, and the number of discrete objects (i.e., collections of things, but without yet using number to enumerate “how many”). The concept of a number is introduced here through the measurement of the quantity, which is carried out through the “deposition” of a unit of measurement on the measured quantity and the calculation of such depositions. The number in this case is a characteristic of the quantity and depends not only on the measured quantity, but also on the chosen measure. By changing the conditions for solving measurement problems and their inverse ones (reproducing a quantity through adding of measurements), students “grow” different types of numbers and ways of designating them (single and multi-digit numbers in different counting systems). As a result, children know for sure that (a) the number always refers to some quantity (when asked “how much?” children would always specify – “what exactly?”), (b) the choice of the measure (“what did they measure?”) is not arbitrary, the measure is always a part of the measured quantity, and (c) the number is needed to preserve/reproduce in new conditions the relationship between the quantity and the measure by which it is measured. And, finally, after the introduction of the concept of number, it is not just “worked out” in various tasks, but is developed into an integral system of learning tasks. It means that the end of Grade 1 a number line appears to be as the general model of number, as special cultural tool. Such curriculum can be considered as completely changing of traditional math education paradigm and it can be called as Relational paradigm instead of Operational paradigm (Polotskaia, 2018).

Studies have shown that certain differences in cognitive and personality characteristics (Shadrikov et al., 2011; Gordeeva et al., 2018) can be detected between DE and TE students. Speaking about mathematics DE students often demonstrate higher levels of mathematical ability than TE students (Atakhanov, 1995; Pavlova, 2008). However, most of these studies measured the differences at the end of Fourth Grade (some researchers, Moxhay, 2008, demonstrated developmental effects after Grade 2), and there are not many which have assessed the effects after the 1st year of the DE curriculum. Since there are such crucial differences between the 1st year mathematics curriculum designed by Davydov and other math programs, it makes sense to compare students in Davydov’s math program after Grade 1 with the same students before completing Grade 1. The other interesting research questions concern the impact of school readiness to development of key math abilities. Is it really true that students with low school readiness cannot achieve good results? According to J. Piaget teaching and learning should adapt to child development while Vygotsky (1978) believed that good teaching leads to development. Is it really true that Davydov’s math curriculum does not required special child school readiness?

## School Readiness

School readiness is not assumed to correspond to a child’s chronological age or specific capabilities; it includes factors such as the child’s level of mental development, as well as preschool factors, such as whether the child participated in some type of high-quality preschool education (Nisskaya, 2018). We are going to speak only about the level of the child’s mental development that is necessary and sufficient for the development of a common school curriculum (Vygotsky, 1956). The main factors determining that level are voluntary behavior regulation and motivation (Gutkina, 2000). Voluntary behavior regulation means following the rules and an adult’s instructions. The construct “voluntary regulation” is very closed to the modern construct “executive functioning.” Executive functioning includes three interrelated components: working memory, inhibitory control, and attention shifting (Miyake et al., 2000) and all of them are considered as predictors of early mathematical learning (Clark et al., 2010; Aarnoudse-Moens et al., 2013). Only two of them (inhibitory control, and attention shifting) can be considered as the essential parts of psychological readiness to school. But it would be good to assess the impact of voluntary regulation to effects after specific Davydov’s curriculum.

Motivation in this context is also called the “inner schoolchild position” – the child’s attitude toward school showing that he or she is willing and ready to fulfill the duties of a student. Motivational readiness is a factor of voluntary behavior development (Gutkina, 2000), but it can be analyzed independently. Some studies have shown that children with high motivational readiness learn better than others (Nezhnova, 1981).

The other essential component of school readiness is intellectual component. According to J. Piaget the most critical here is preservation. The concept of “preservation” means that an object or a set of objects are recognized as unchanged in the composition of the elements or in any physical parameter, despite changes in their shape or external location. At the preoperative stage of the development of intelligence (Piaget, 1964), the child relies exclusively on perceptual visualization, therefore, any movement of elements within a set means for him a change in the set itself as a whole. At the level of specific operations (usually, at the beginning of Grade 1) an understanding of the principle of preservation in relation to different physical characteristics of objects and phenomena (mass, weight, length, etc.) does not arise in a child simultaneously (i.e., understanding of the principle of conservation of mass does not mean that understanding of the principle of preservation of weight and volume). But the level of preservation (how many particular characteristics student have already preserved) can differentiate students according their cognitive readiness. We choose the ability to preserve also because is strongly depended on understanding what we are going to measure and there was some findings (see, for example, Obukhova, 1972), which have shown that after special teaching (teaching how to compere objects, using different characteristics through measurement) even 6-year old children can have logical preservation. The content of Davydov’s math curriculum includes very same things, so we expected that the level of logical preservation does not play very important role in children’s improvements.

## Possible Developmental Effects of Davydov’s Math Curriculum

In exploring the developmental effects of Davydov’s curriculum, Moxhay (2008) used specific problems for assessment, the very same ones which are used in real lessons (for example, telling the child to cut off a piece of paper tape “just like this one,” but not allowing him to carry it from one table to the other). Moxhay also posed problems to the children, which are not specific to a math curriculum, but measure general theoretical thinking (Zak, 1984). In our opinion, it is interesting to compare characteristics, which are closely connected with the contents of Davydov’s math curriculum, especially after Grade 1 (so, they should be some math abilities). We have chosen two groups of characteristics: (1) the general ones (which can improve during the year regardless of the specific Davydov’s program) and (2) the specific ones (which probably can be the result of Davydov’s curriculum). As the general characteristics, we have chosen, first of all, ability to compare objects using only one characteristic in spite of others (*comparison*). Comparison is the part of preschool education in most kindergartens in Russia (Veraksa et al., 2019) and it can improve after a 1 year because almost all math existing programs for Grade 1 include tasks about comparison. They should be able to highlight the comparison parameter before making the comparison itself, and not get confused if the objects are also similar in other aspects. The second characteristic is the measurement ability (it was ability to measure of length as the simplest one) – to start with the beginning, to choose the same units and to put them one by one without any spaces (*measurement*). The ability to measure of length is often used in real life and also a part of preschool education curriculum, so such tasks can be solved and we have some reasons to expect improvements here too. The last general ability is the ability to solve simple problems of addition and subtraction (*math problems*; many students can do it before the Grade 1 and it also can improve during the year).

Also, we decided to check some other specific abilities, which should be the possible results of specific Davydov’s curriculum. The first one is the ability to put numbers on a number line (*number line*) and the second one is the ability to measure quantity (*measuring of quantity*; in our case – square) in case quantity and the different units are suggested, so children are supposed to find an exact number. This was necessary to show the extent to which students had mastered the program as a whole.

What were our initial expectations?

## The Hypotheses

First, we expected that the improvements in general math abilities probably would not depend on initial levels of voluntary regulation, motivation, and preservation. This is because the main goal of the Davydov’s curriculum is *developmental* education, which probably is suitable for all children regardless of their initial school readiness. Such curriculum should lead to the increasing of motivation and voluntary regulation and also preservation (because during the Grade 1 children work constantly with different physical characteristics of objects and phenomena).

General Hypothesis 1: Improvements in general math abilities after the curriculum do not depend on initial level of voluntary regulation, motivation, and preservation.
Hypothesis 1.1: Improvements in general math abilities (comparison, measurement, and math problems) do not depend on level of *voluntary regulation*.
Hypothesis 1.2: Improvements in general math abilities (comparison, measurement, and math problems) after the curriculum do not depend on level of *motivation*.
Hypothesis 1.3: Improvements in general math abilities (comparison, measurement, and math problems) after the curriculum do not depend on level of *preservation*.
General Hypothesis 2: There are not any differences in specific math capabilities (measuring of quantity and number line) after the year’s curriculum between students with different levels of voluntary regulation, motivation, and preservation.
Hypothesis 2.1: There are not any differences in specific math capabilities among students with different levels of voluntary regulation.
Hypothesis 2.2: There are not any differences in specific math capabilities among students with different levels of motivation.
Hypothesis 2.3: There are not any differences in specific math capabilities among students with different levels of preservation.
General Hypothesis 3: There are not any differences between classes with different in teacher experience. We did not expect any differences between classes with teachers with different levels of experience. The main reason of that is that both teachers did not have any experience of teaching according the Elkonin-Davydov’s principals.

## Materials and Methods

### Participants

The participants were 46 first graders from one public school in Moscow, 21 boys and 25 girls (average age = 7.5, *SD* = 0.46). The participants were drawn from two school classes with different teachers (class A: *N* = 23, and class B: *N* = 23). All participants studied mathematics according Davydov’s mathematics curriculum, but their teachers had different levels of experience. The teacher of class A had worked at the school more than 25 years, while the teacher of class B had just graduated from university before taking this class. However, it was the first experience for both teachers to teach according to the principles of the Elkonin-Davydov’s system.

### Materials

#### School Readiness

##### Motivational Readiness

To measure motivational readiness, we used the “A conversation about school” method designed by Nezhnova (1981). The conversation aims at assessing the development of the “inner schoolchild position.” We suggested five alternatives to the children and asked them which they preferred. The children received a piece of paper with red and blue circles drawn to represent red and blue schools, respectively. Each question had a description of a “red” or “blue” school, and the children had to choose the school they liked more and mark the corresponding circle.

For each answer corresponding to a positive inner schoolchild position, the children received a 1; otherwise they got a 0. The questions concerned: (1) the lesson schedule (in the red school, you will study more reading, mathematics, and writing, and less drawing, physical education, and music; in the blue one, you will study more drawing, physical education, and music, and less reading, mathematics, and writing); (2) school rules (in the red one, there are a lot of rules, while in the blue, no rules at all); (3) studying in school or at home; (4) the possibility of inviting mothers to be schoolteachers; and (5) the method of assessment (marks in the red school and sweets for performance in the blue one). The maximum score for that test was 5.

##### Voluntary Regulation

To measure voluntary regulation, we used the “Graphic dictation” test designed by D.B. Elkonin (Gutkina, 2000, p. 69). The children are invited to draw on the checkered paper a line down, then right, up, right, down, and so on. In this case, the test administrator should not dictate where the line should end, and once the pattern of organization is identifiable, should invite the students to continue the picture themselves. We used one pattern for training and three different patterns for the test. Each of them was assessed from 0 to 4. The maximum score for that test was 12.

##### Preservation

**Figure 1:**
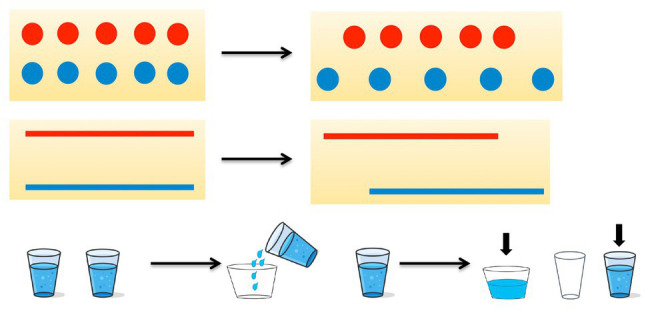
The initial pictures and changed pictures, which were shown to students for preservation measurement.

We measured preservation using J. Piaget’s tests. Children were presented with initial pictures and changed pictures (see Figure 1). They were then asked to make sure that the number of circles (length of strips or amounts of water) was the same. Then the administrator pointed out that something had changed (blue circles were pushed apart, a blue strip was moved, water from one glass was poured into another glass). Then he/she asked which picture had more circles now; which strip was longer; which glass held more water, or maybe they were the same? The children had to circle those which were more, or put a sign “=” if they were equal.

#### Developmental Effects of the Math Curriculum

##### General Math Abilities

###### Comparison of Objects

**Figure 2:**
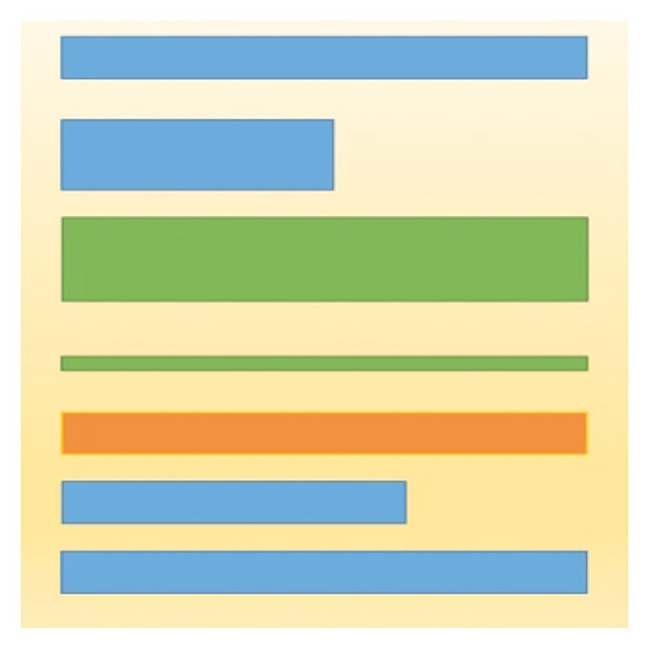
The task of assessing the ability to compare objects by one characteristic. Students were supposed to circle those strips that were equal in length.

To measure this skill, we offered a picture with seven strips (see Figure 2) and asked the children to circle those that were equal in length. Moreover, among the strips there were some not only of the same length, but also of the same color and width, or of the same color, width, and length at the same time. In total, there were five rectangles of equal length. For this task the maximum score was 5.

###### Measurement

**Figure 3:**
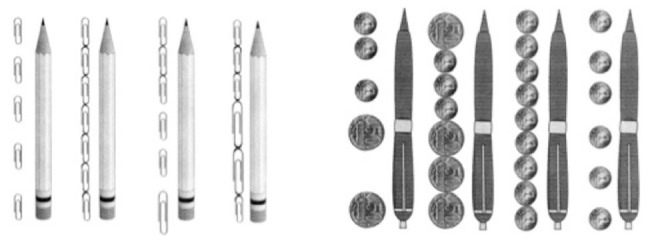
Two tasks to measure the ability to choose an appropriate measure of quantity.

We suggested a picture and the instruction was as follows: “The children measured the pencil with paper clips. Here’s how they did it. Which of them measured the pencil correctly? Please circle.” After that we gave them the next picture (with pens measured by coins) and the same instruction (see Figure 3).

###### Math Problems

There were five simple problems (1 + 3, 7–5, 9 + 1, 2 + 6, and 8–4); the maximum score was 5.

##### Specific Math Capabilities

**Figure 4:**
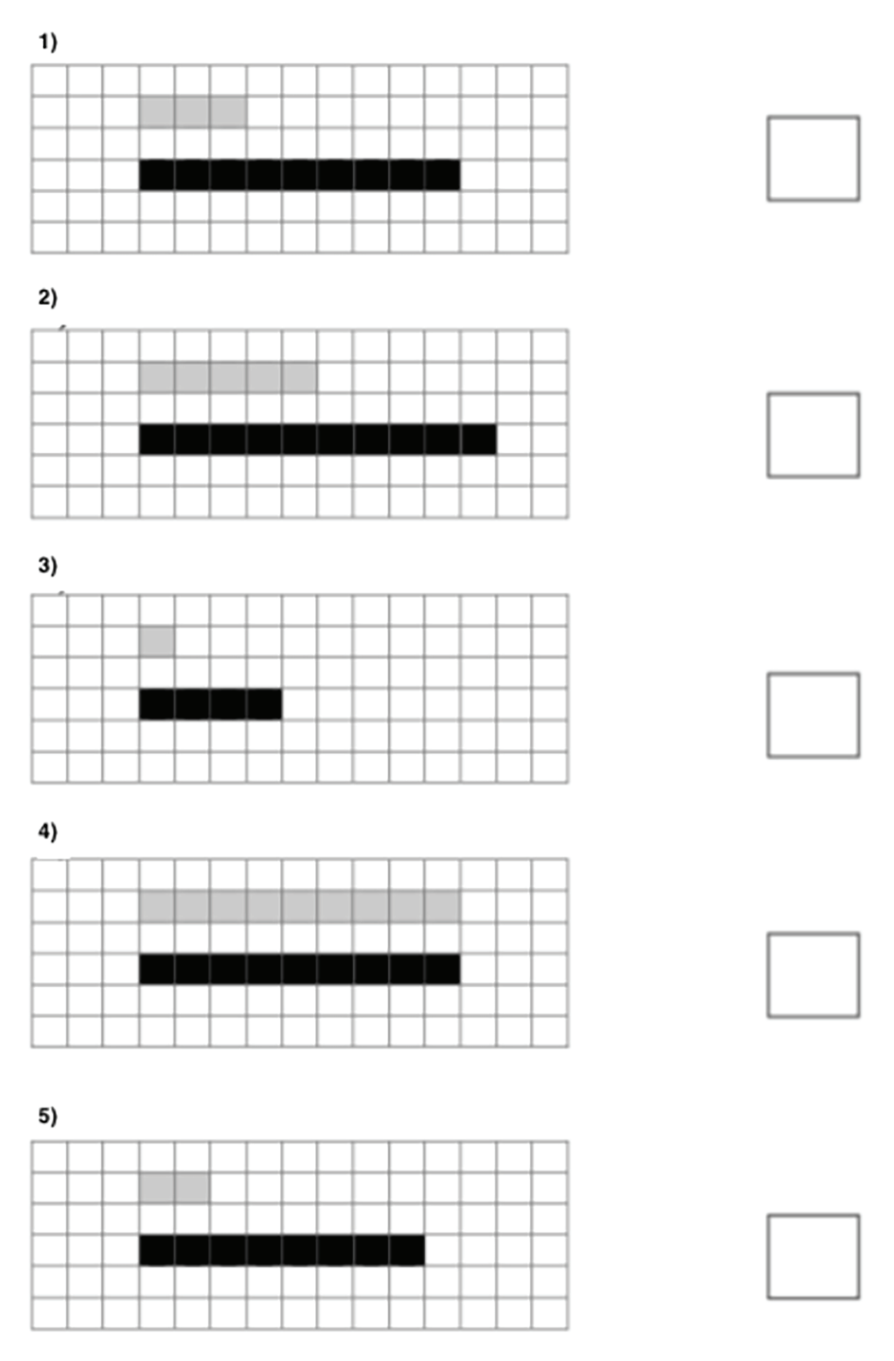
Tasks for assessing the ability to measure quantity using different measures.

For specific math effects, we assessed two abilities. The first one was the ability to measure quantity using different measures (*measuring of quantity*). Students had to supply a number describing how many times a small measure fits into a large measure (see Figure 4).

The second measured ability was the ability to put numbers on a number line (*number line*). We used these standard problems: (1) the number line was directed to the right; (2) a number line was directed to the left; (3) there was a number line in which the measure was one cell and several cells; and (4) instead of a number line, there was a line without direction with a single digit. The last problem had no solution.

External validity was provided by the teacher’s assessment of the same parameters. We asked teacher to assess all involved students using same characteristics: the general characteristics (comparison, measurement, math problems) and the specific ones (measuring of quantity and number line). All correlations were significant (*p* < 0.05, Pirson’ correlation), except comparison – it was not. Probably, it can be explained by the fact that teacher assess in more detail (not only lengh comparison but also comparison of volume, square, and others).

### Design

The study included two stages. At the beginning of the first school year, we measured the children’s motivation, voluntary regulation, and general math capabilities. At the beginning of the second school year, we measured general math abilities again, as well as specific math abilities.

### Data Processing

We used SPSS Statistics 23 for data processing.

## Results

Descriptive statistics are presented in Table 1. The results showed that all the children became better in all the general math abilities – comparison, measurement, and math problems (we have used Related Samples Wilcoxon Signed Rank test, *p* < 0.001). Moreover, such improvements in comparison, measurement, and math problems are significant for each groups of respondents (we have made One-Sample *t*-test for all three regulation groups, motivation groups, and preservation groups, *p* < 0.001 (only for low and high levels of motivation improvement in measurement significant with *p* < 0.01).

**Table 1:** Descriptive statistics for all measured characteristics.

	*N*	Minimum	Maximum	Mean	Std. deviation	Variance
**School readiness**						
Voluntary regulation (max 16)	40	0	16	11.3	4.02046	16.164
Motivation (max 5)	36	1	5	3.6944	1.1909	1.418
Preservation (max 3)	44	0	3	2.5227	0.82091	0.674
**General math effects (1 – before the curriculum, 2 – after the curriculum)**						
Measurement 1	43	0	2	1.1628	0.78468	0.616
Measurement 2	39	0	2	1.7692	0.48458	0.235
Comparison 1	44	0	5	3.7727	1.3954	1.947
Comparison 2	39	0	5	3.9231	1.30555	1.704
Math problems 1	40	0	5	4.075	1.28876	1.661
Math problems 2	39	4	5	4.8205	0.38878	0.151
**Specific math effects**						
Measuring of quantity (max 5)	37	0	5	2.1	2.01	4.04
Number line (max 4)	37	0	2	0.94	0.66	0.44

We have not found any significant differences between the two classes in voluntary regulation, motivation, preservation, measurement, and math tasks at the beginning of the first school year except differences in the tasks of comparison; class A did it better (*p* < 0.01, Mann-Whitney test for independent samples). We also did not explore any correlations between voluntary regulation, motivation, or preservation, so we can divide the whole sample into groups separately by each these characteristics. How we did it? We divided all participants into three groups: low (25th percentile and below), average (26th–74th percentile), and high levels (75th percentile and above), depending on their (a) motivational readiness and (b) regulation readiness. Concerning preservation we have found that most children did the preservation task pretty perfect (65.2%), so we decided to divide the whole sample into two groups – high level and lower levels. The distribution of the sample depending on voluntary regulation, motivation, and preservation is presented in Table 2.

**Table 2:** The distribution of the sample depending on voluntary regulation, motivation, and preservation (in %).

	Low level	Medium level	High level	Missing
Voluntary regulation	35	28.1	23.9	13.0
Motivation	30.4	23.9	23.9	21.7
Preservation	30.4		65.2	4.4

**Figure 5:**
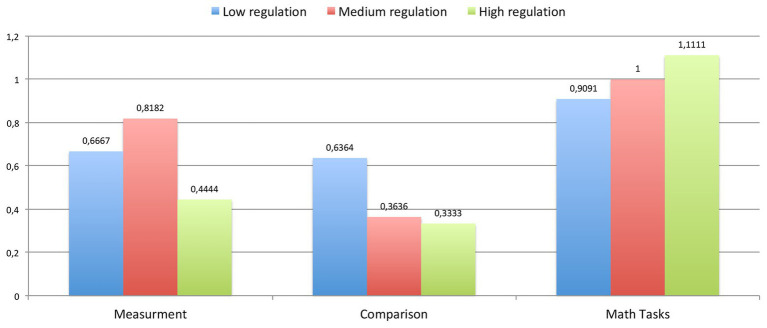
The average improvements in each characteristic (measurement, comparison, and math problems) for each group of children (with low, medium, and high regulation readiness).

**Figure 6:**
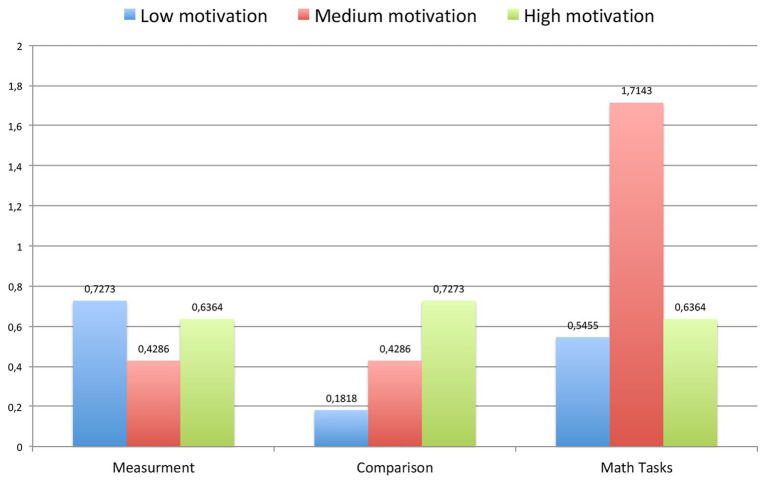
The average improvements in each characteristic (measurement, comparison, and math problems) for each group of children (with low, medium, and high motivational readiness).

**Figure 7:**
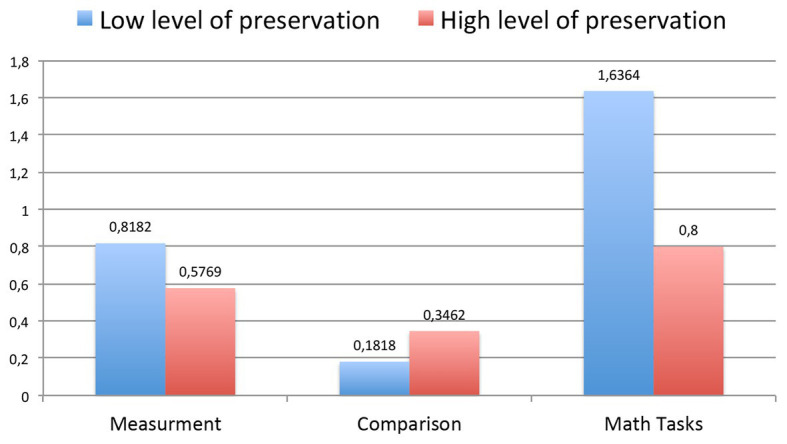
The average improvements in each characteristic (measurement, comparison, and math problems) for each group of children (with low and high levels of preservation).

Also, for each participant, we have counted improvements in all three general math abilities among whole sample as the difference between levels after a year and before the year (you can see in Table 3, how many students have improved each ability). Improvements of each group depending on voluntary regulation, motivational readiness, and preservation are shown on the pictures (Figures 5–7).

**Table 3:** The distribution of the sample depending on improvement in general math abilities.

	No improvements	Improvements	Missing
Comparison (*N* = 37)	23 (50.1%)	14 (30.5%)	9 (19.6%)
Measurement (*N* = 37)	19 (41.3%)	18 (39.1%)	9 (19.6%)
Math tasks (*N* = 36)	19 (41.3%)	7 (37.1%)	10 (21.6%)

We also made correlation between regulation readiness with all general math abilities after a year and the same for motivational readiness and for preservation. We results showed that regulation readiness positively correlates with measurement (*R* = 0.39, *p* < 0.05, *N* = 33) and with math tasks (but only according to teacher’s assessment; *R* = 0.35, *p* < 0.05). We have not founded any correlations for motivational readiness. But preservation positively correlates with measurement and comparison, assessed by teacher. So, there are some reasons to think that regulation readiness and logical preservation are more important for Davydov’s curriculum than motivational readiness. The ANOVA analysis showed the interaction of regulatory readiness and preservation in student’s progress in the ability to measure.

We then proceeded to test all our hypotheses.

General Hypothesis 1 was completely confirmed. Improvements in general math abilities after the curriculum do not depend on initial level of voluntary regulation, motivation, and preservation.

Hypothesis 1.1 was confirmed. Improvements in general math abilities (comparison, measurement, and math problems) do not depend on level of *voluntary regulation* (*t*-test for independent samples).

Which particular regulation groups have significantly improved their general math abilities comparing with overs? According to our results, all groups of students became significantly better in all math abilities (*p* < 0.001, One-sample *t*-test).

Hypothesis 1.2 was confirmed. Improvements in general math abilities (comparison, measurement, and math problems) after the curriculum do not depend on level of *motivation*. Interesting, that children with medium level of motivation have showed the most improvements in math tasks comparing with children with high level of motivation (differences are not significant, but they can be if we have more respondents, because *p* = 0.08).

Hypothesis 1.3 was confirmed. Improvements in general math abilities (comparison, measurement, and math problems) after the curriculum do not depend on level of preservation.

The second, we expected that there could not be differences in specific math effects between children with different levels of motivation, voluntary regulation, and preservation after the year’s curriculum.

General Hypothesis 2 *was partly confirmed*. There are not any differences in specific math capabilities (measuring of quantity and number line) after the year’s curriculum between students with different levels of voluntary regulation, motivation, and preservation.

Hypothesis 2.1 was confirmed. There are not any differences in specific math capabilities among students with different levels of voluntary regulation. There was some tendency that there were differences between groups with high and low levels of voluntary regulation because according to teacher’s assessment children with high level are better in line number (*p* = 0.067) and in measuring of quantity (*p* = 0.07).

Hypothesis 2.2 was partly confirmed. There are not any differences in specific math capabilities among students with different levels of motivation except measuring of quantity (we explored that students with low motivation did it better after a year of learning than students with high level of motivation). But this fact does not confirm by the results of teacher’s assessment (according to teacher there are not any differences in measuring of quantity between low and high groups).

Hypothesis 2.3 was confirmed. There are not any differences in specific math capabilities among students with different levels of preservation.

General Hypothesis 3 was confirmed. There are not any differences between classes with different teacher’s experience. But there are some reasons to think that class A, with the less experienced teacher, was better (*p* = 0.07) in measuring quantity (only according to teacher’s assessment).

## Discussion

Davydov’s curriculum is often considered by parents (and some teachers) to be more difficult than other math curricula. The program has special requirements not so much related to the child’s capabilities as to the quality of the child’s psychological readiness for school. Our study involved children with different levels of motivational readiness for school, with different levels of voluntary regulation, and different levels of logical preservation. After a year of study under the Davydov’s curriculum, there were changes in general math abilities for all groups of children (from 30 to 40% of whole sample have improved their abilities), and such changes, according to our results do not depend on their initial levels of school readiness. We can see that the progress is possible for children with all levels of voluntary regulation, motivational, and cognitive readiness. So, the Davydov’ program, probably, does not have any special requirements, related to the quality of the child’s psychological readiness for school, which give us a reasons to think that Vygotsky’s point about interconnection between learning and development (Vygotsky, 1956) was correct. Especially it concerns the level of logical preservation in terms of J. Piaget – according to our results it does not play an essential role. Works of L. Obujkova, actually, confirm this results because she has shown that logical preservation can be acquired through measurement (Obukhova, 1972). We also did not found any differences in specific math effects between classes with teachers with different levels of experience, but it can be explained by the fact that both teachers did not have the experience in teaching according to Davydov principals. We suppose, more effective would be to analyze and compare the real lessons of each teacher in terms of compliance with the Cultural Historical Activity Theory (CHAT) methodology. Let us discuss several possible limitations to our study. First, in general the effectiveness of classroom methods of school readiness’ diagnostics is always lower than the effectiveness of methods conducted individually with a child. Unfortunately, due to the COVID-19 pandemic, we were not able to conduct individual examinations. So in future it would be good to test our respondents individually. Secondly, the teachers’ lack of experience with the Elkonin-Davydov program could mean that the program itself was not being implemented quite correctly. This means that the effects obtained cannot be reliably recognized as the effects of this educational system. And, finally, an insufficient number of subjects could also affect the results. Nevertheless, we believe that these results reflect reality to some extent, although they require further research.

## Conclusion

In our research, we have analyzed some developmental effects of the realization of Davydov’s math curriculum in Grade 1, in relation to the children’s levels of school readiness (motivation and voluntary regulation) and their teacher’s experience. First, we expected to find differences in general math capabilities at the beginning of the Grade 1 between particular groups of students. Our first general hypothesis was completely confirmed: improvements in general math abilities after the curriculum do not depend on initial level of voluntary regulation, motivation, and logical preservation. Our second general hypothesis was partly confirmed. There are not any differences in specific math capabilities (measuring of quantity and number line) after the year’s curriculum between students with different levels of voluntary regulation, motivation, and preservation, but results are contradictable: according to teacher’s assessment there are differences in measuring of quantity between low and high motivated children, but according to our methods students with low motivation did it better than students with high level of motivation!. Our third hypothesis was confirmed. There are not any differences between classes with different teacher’s experience. So, the most interesting our finding was school readiness (understanding as motivational, voluntary regulation, and cognitive) do not play so important role in teaching mathematics, based on Elkonin-Davydov principals.

## Data Availability Statement

The raw data supporting the conclusions of this article will be made available by the authors, without undue reservation.

## Ethics Statement

The studies involving human participants were reviewed and approved by Ethics committee of the Russian Psychological Society. Written informed consent to participate in this study was provided by the participants’ legal guardian/next of kin.

## Author Contributions

AS designed the model and the computational framework, picked up and analyzed the data, and wrote this manuscript.

### Conflict of Interest

The author declares that the research was conducted in the absence of any commercial or financial relationships that could be construed as a potential conflict of interest.
